# Predictability of Liver-Related Seromarkers for the Risk of Hepatocellular Carcinoma in Chronic Hepatitis B Patients

**DOI:** 10.1371/journal.pone.0061448

**Published:** 2013-04-17

**Authors:** Yu-Ju Lin, Mei-Hsuan Lee, Hwai-I Yang, Chin-Lan Jen, San-Lin You, Li-Yu Wang, Sheng-Nan Lu, Jessica Liu, Chien-Jen Chen

**Affiliations:** 1 Institute of Microbiology and Immunology, School of Life Sciences, National Yang-Ming University, Taipei, Taiwan; 2 Genomics Research Center, Academia Sinica, Taipei, Taiwan; 3 Institute of Clinical Medicine, National Yang-Ming University, Taipei, Taiwan; 4 Graduate Institute of Clinical Medical Science, College of Medicine, China Medical University, Taichung, Taiwan; 5 Molecular and Genomic Epidemiology Center, China Medical University Hospital, Taichung, Taiwan; 6 School of Medicine, MacKay Medical College, Taipei, Taiwan; 7 Department of Internal Medicine, Kaohsiung Chang-Gung Memorial Hospital, Kaohsiung, Taiwan; 8 Graduate Institute of Epidemiology and Preventive Medicine, College of Public Health, National Taiwan University, Taipei, Taiwan; Yonsei University College of Medicine, Republic of Korea

## Abstract

**Background:**

Hepatitis B virus (HBV)-related hepatocellular carcinoma (HCC) is a major global health problem. A few risk calculators have been developed using mainly HBV seromarkers as predictors. However, serum HBV DNA level, HBV genotype, and mutants are not routinely checked in regular health examinations. This study aimed to assess the predictability of HCC risk in chronic hepatitis B patients, using a combination of liver-related seromarkers combined with or without HBV seromarkers.

**Methods:**

A prospective cohort of 1,822 anti-HCV-seronegative chronic HBV carriers was included in this study. Liver-related seromarkers including aspartate aminotransferase (AST), alanine aminotransferase (ALT), alpha-fetoprotein (AFP), gamma-glutamyltransferase (GGT), total bilirubin, total protein, albumin, serum globulins, apolipoprotein A1, and apolipoprotein B were examined. Hazard ratios of HCC with 95% confidence intervals were estimated using Cox proportional hazards regression models. Regression coefficients of seromarkers significantly associated with HCC risk in multivariate analyses were used to create integer risk scores. The predictability of various risk models were assessed by area under receiver operating characteristic curves (AUROCs).

**Results:**

During a median follow-up of 5.9 years, 48 newly-developed HCC cases were ascertained. Elevated serum levels of ALT (≥28 U/L), AFP (≥5 ng/mL), and GGT (≥41 U/L), an increased AST/ALT ratio (AAR, ≥1), and lowered serum levels of albumin (≤4.1 g/dL) and alpha-1 globulin (≤0.2 g/dL) were significantly associated with an increased HCC risk (P<0.05) in multivariate analysis. The risk model incorporating age, gender, AAR, and serum levels of ALT, AFP, GGT, albumin, and alpha-1 globulin had an AUROC of 0.89 for predicting 6-year HCC incidence. The AUROC was 0.91 after the addition of HBV seromarkers into the model, and 0.83 for the model without liver-related seromarkers, with the exception of ALT.

**Conclusion:**

Liver-related seromarkers may be combined into useful risk models for predicting HBV-related HCC risk.

## Introduction

Chronic hepatitis B virus (HBV) infection is one of the major causes of deaths from end-stage liver diseases. The lifetime risk (30–78 years of age) of developing cirrhosis and hepatocellular carcinoma (HCC) was estimated to be as high as 41.5% and 21.7%, respectively. [1] Chronic HBV infection accounts for around 50% of total HCC cases and virtually all childhood HCC cases. [2] Even though the prevalence of chronic HBV infection is gradually decreasing in most regions due to the implementation of HBV vaccination programs, it remains a serious public health problem worldwide. An updated statistic estimates that 240 million people are chronically infected with HBV in the world, with the highest burden lying in developing countries with inadequate medical resources such as sub-Saharan Africa and parts of Eastern Asia. [3].

A number of risk predictors for HBV-associated HCC have been identified in previous studies.[4]–[10] They include older age, male gender, family history of HCC, elevated serum level of alanine aminotransferase (ALT), alcohol drinking, cirrhosis status, hepatitis B e antigen (HBeAg) seropositivity, high HBV DNA viral load, HBV genotype C, and the HBV core promoter mutation. In addition, a few nomograms and risk scores have also been proposed in recent years to facilitate the identification of high-risk chronic hepatitis B patients for anti-viral treatment and screening of HCC. [11]–[14] Among the proposed risk models, serum HBV DNA level is an important HCC predictor. Continuous anti-viral treatment of patients with chronic hepatitis B has significantly reduced the subsequent development of hepatic decompensation and HCC. [15]–[17] Thus, prediction models may help chronic HBV carriers to be more conscious of the severity of their disease.

However, some HBV seromarkers included in previous risk calculators, such as HBV viral load, genotype, and mutants, are not routinely tested in regular health examinations. On the other hand, Liver-related seromarkers such as aspartate aminotransferase (AST), ALT, alpha-fetoprotein (AFP), total bilirubin, total protein, albumin, alpha-1 globulin, alpha-2 globulin, beta globulin, gamma globulin, gamma-glutamyltransferase (GGT), apolipoprotein A1 (apoA1), and apolipoprotein B (apoB) are frequently tested in clinical practice to assess liver injury, inflammation, fibrosis, and dysfunction. However, their capability to predict subsequent HCC risk has not been well assessed. [18], [19].

This study aimed to assess the ability of liver-related seromarkers to predict HCC risk in chronic hepatitis B patients, both without, or in combination with HBV seromarkers. Several HCC risk prediction models with high validity were developed.

## Methods

### Study Participants

**Figure 1:**
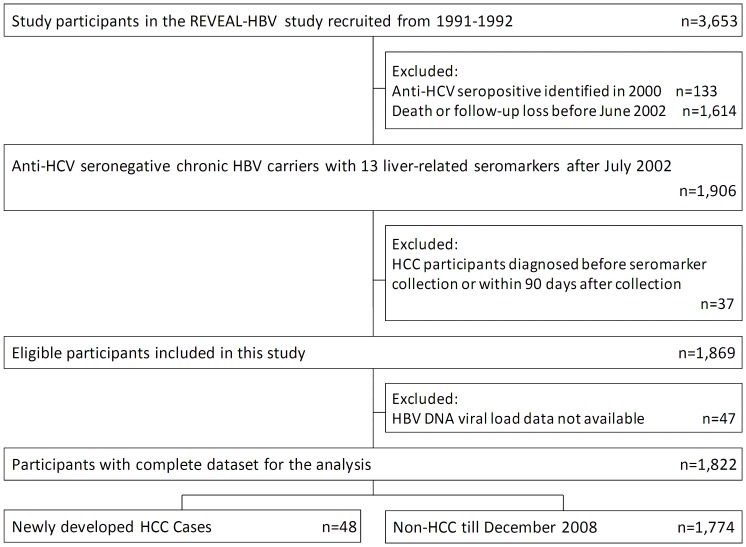
Flowchart of study participants selected from original REVEAL-HBV cohort and included in this analysis.

A sub-cohort derived from the R.E.V.E.A.L.-HBV Study was included in this analysis. The enrollment of the R.E.V.E.A.L.-HBV cohort has been described previously. [4], [13], [20] Briefly, it was a community-based cohort of participants seropositive for hepatitis B surface antigen (HBsAg) and seronegative for antibodies against hepatitis C virus (anti-HCV). Participants were enrolled from seven townships in Taiwan during 1991–1992 after obtaining each participant's informed consent. In addition to HBV seromarkers and serum ALT levels, a total of 12 liver-related seromarkers including AST, AFP, GGT, total bilirubin, total protein, albumin, alpha-1 globulin, alpha-2 globulin, beta globulin, gamma globulin, apoA1, and apoB were included in follow-up examination starting in July, 2002. The date each participant’s first examination of liver-related seromarkers was defined as the entry date in this analysis. Only participants with complete seromarker data and without new HCV infections were included in this study. Furthermore, participants who developed HCC before or within 3 months after their first examination of liver-related seromarkers were excluded from this study. A total of 1,822 chronic HBV carriers (HBsAg-seropositive for more than 6 months) were included in this analysis (Figure 1).

### Questionnaire Interview, Ascertainment of Cirrhosis, and Blood Collection

All participants were personally interviewed by well trained public health nurses using a structured questionnaire. Information on sociodemographic characteristics, dietary intake, habits of cigarette smoking and alcohol consumption, personal medical and surgical history, and family history of cancers and other major diseases was collected from each participant. Liver cirrhosis was diagnosed by high-resolution real-time ultrasound, based on a quantitative scoring system derived from the appearance of the liver surface, liver parenchymal texture, intrahepatic blood vessel size, and splenic size. All ultrasounds were performed and interpreted according to a standardized protocol. Computerized data linkage with National Health Insurance profiles in Taiwan was used to ensure the complete ascertainment of cirrhosis cases. [20] Cirrhosis status was defined as cirrhosis diagnosed before or at the time of each participant’s first examination of liver-related seromarkers.

### Laboratory Examinations

A 10-mL blood sample was collected from each participant during follow-up health examinations. Fractionated aliquots of serum samples were frozen at −70°C before tested. Serum samples were tested for a battery of 13 seromarkers including AST, ALT, AFP, total bilirubin, total protein, albumin, alpha-1 globulin, alpha-2 globulin, beta globulin, gamma globulin, GGT, apoA1, and apoB, in addition to HBV seromarkers such as serum HBV DNA level and HBeAg serostatus. AST, ALT, total bilirubin, total protein, GGT, apoA1, and apoB were tested by autoanalyzer (Toshiba TBA-200FR, Japan) with commercial reagents (Denka Seiken, Tokyo, Japan). AFP was measured by radioimmunoassay with the BRAHMS AFP KRYPTOR (Brahms France, Sartrouville, France). Albumin, alpha-1 globulin, alpha-2 globulin, beta globulin, and gamma globulin were analyzed using protein electrophoresis (Helena Lab., TX, USA).

Seromarkers of chronic HBV and HCV infection were examined using commercial kits: HBeAg and HBsAg by radioimmunoassay (Abbott Laboratories, North Chicago, IL), and anti-HCV by enzyme-linked immunoassay using second-generation test kits (Abbott Laboratories). Serum HBV DNA level was quantified using a polymerase chain reaction–based nucleic acid amplification test (Cobas Amplicor HBV monitor test kit; Roche Diagnostics, Indianapolis, IN) with a certified detection limit of 300 copies/mL.

### Ascertainment of Newly-developed Hepatocellular Carcinoma

Every participant was tested by abdominal ultrasonography every 3–12 months. Suspected HCC cases identified in regular health examinations were referred for further examinations using liver biopsy, angiogram, or computed tomography. Newly-developed HCC cases were ascertained through computerized linkage with national cancer registration and death certification profiles. The diagnostic criteria included histopathological confirmation, two coincident imaging findings, or AFP levels ≥400 ng/mL plus one positive imaging finding. [21].

### Statistical Analysis

All statistical analyses were performed with SAS version 9.2 (SAS Institute, Cary, NC). Hazard ratios (HR) with 95% confidence intervals (CI) were derived to assess associations between predictors and newly-developed HCC through Cox proportional hazards regression analyses after adjustment for age, gender, and residential township. All 13 seromarkers were dichotomized into binary data using either upper or lower quartiles of the seromarkers in all participants as the cut-off points. The cut-off point for the AST/ALT ratio (AAR) was defined as 1 according to a previous study. [22] During the derivation of risk models, only seromarkers significantly associated with newly-developed HCC in multivariate regression models were included. The correlations among predictors included in the final scoring systems were evaluated by Pearson correlation coefficients.

The risk score for each 5-year increment in age was set as 1. Regression coefficients for other predictors included in the regression model were converted into integer scores by rounding the quotients of dividing each regression coefficient by the regression coefficient for each 5-year increment in age to the nearest integer. Sum scores were derived by adding the scores assigned to all risk predictors included in the specific model. The validity of risk models for the 6-year prediction of HCC risk was evaluated by using the area under the receiver operating characteristic curve (AUROC), the best Youden index (sensitivity+specificity-1), the positive likelihood ratio [sensitivity/(1-specificity)], and the negative likelihood ratio [(1-sensitivity)/specificity] [23]. For evaluating and comparing the AUROCs of different prediction models, a nonparametric comparison of areas under correlated ROC curves was used. [24] The estimated 6-year cumulative incidence of HCC was calculated according to the following equation:
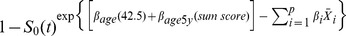
where *S*_0_(t) is the estimate of the average survival at 6 years, 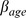 is the regression coefficient of age, 42.5 is the age value we considered as the base, 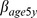 is the regression coefficient for each 5-year increment in age, 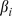 is the regression coefficient for the ith covariate, and 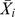 is the mean level of the ith covariate. [13], [14], [25] The Student's t-test was used for comparing mean total scores between HCC and non-HCC groups among cirrhotic patients, and between HCC participants with and without cirrhosis. The paired t-test was used for testing the stability of sum scores in model II.

### Ethics Statement

This study was approved by the Institutional Review Board of the College of Public Health, National Taiwan University in Taipei, Taiwan.

## Results

### Risk Predictors of Hepatocellular Carcinoma

During the follow-up of 1,822 anti-HCV-seronegative chronic HBV carriers for a median period of 5.9 years (range, 0.3–6.5 years), 48 newly-developed HCC cases occurred. HCC incidence rates stratified by risk predictors including age, gender, cirrhosis status, HBV seromarkers of serum HBV DNA level and HBeAg serostatus, the 13 liver-related seromarkers, and AAR are shown in Table 1. A significantly increased HCC incidence was associated with older age, male gender, cirrhosis, serum HBV DNA levels >10,000 copies/mL, and HBeAg seropositivity. Elevated serum levels of AST, ALT, AFP, GGT, and gamma globulin, increased AAR, and lower serum levels of albumin and alpha-1 globulin were also significantly associated with increased HCC risk. In the multivariate analysis, 6 seromarkers including ALT, AAR, AFP, GGT, albumin, and alpha-1 globulin remained significantly associated with the risk of HCC, and were included in the models for the prediction of the 6-year risk of HCC. Serum levels of AST and gamma globulin were no longer significantly associated with HCC risk after adjustment for other HCC predictors in the multivariate analysis. Since ALT and AST were highly correlated with a Pearson correlation coefficient of 0.80, ALT and AST were included into separate multivariate regression analyses. However, AST was still not significantly associated with HCC risk after adjustment for other HCC predictors, even when both ALT and AAR were excluded. The correlations between AAR and ALT (or AST) were low, showing a Pearson correlation coefficient of −0.17 between AAR and ALT, and 0.23 between AAR and AST.

**Table 1:** Incidence Rates and Adjusted Hazard Ratios of Developing Hepatocellular Carcinoma (HCC) by Risk Predictors in 1,822 Anti-HCV-Seronegative Chronic HBV Carriers.

Baseline Demographicor Characteristic^+^	Number (%) ofParticipants(n = 1,822)	Person-yearsof Follow-up(Total = 10,083)	Number ofHCC cases(n = 48)	Incidence Rateper 100,000Person-years	Adjusted Hazard Ratio* (95% confidenceinterval)	P-value
Age						
40–49	595 (32.7)	3,435	5	146	1.00	
50–59	540 (29.6)	3,039	12	395	2.70 (0.95–7.66)	0.062
60+	687 (37.7)	3,609	31	859	6.04 (2.35–15.55)	<0.001
Gender						
Female	407 (22.3)	2,235	6	268	1.00	
Male	1,415 (77.7)	7,848	42	535	2.62 (1.07–6.41)	0.035
Cirrhosis						
No	1,660 (91.1)	9,227	21	228	1.00	
Yes	162 (8.9)	856	27	3,154	12.55 (7.06–22.33)	<0.001
HBV seromarkers						
HBeAg negative and HBVDNA ≤10,000 copies/mL	1,251 (68.7)	7,024	12	171	1.00	
HBeAg negative and HBVDNA >10,000 copies/mL	448 (24.6)	2,404	19	790	5.99 (2.87–12.51)	<0.001
HBeAg positive	123 (6.8)	655	17	2,595	20.71 (9.64–44.50)	<0.001
Serum AST level						
<30 U/L	1,336 (73.3)	7,464	11	147	1.00	
≥30 U/L	486 (26.7)	2,619	37	1,413	9.56 (4.87–18.77)	<0.001
Serum ALT level						
<28 U/L	1,324 (72.7)	7,333	18	245	1.00	
≥28 U/L	498 (27.3)	2,750	30	1,091	5.83 (3.19–10.65)	<0.001
AAR						
<1	651 (35.7)	3,732	4	107	1.00	
≥1	1,171 (64.3)	6,351	44	693	5.78 (2.06–16.24)	0.001
Serum AFP level						
<5 ng/mL	1,390 (76.3)	7,814	15	192	1.00	
≥5 ng/mL	432 (23.7)	2,269	33	1,454	8.16 (4.42–15.07)	<0.001
Serum GGT level						
<41 U/L	1,353 (74.3)	7,600	14	184	1.00	
≥41 U/L	469 (25.7)	2,483	34	1,369	7.73 (4.09–14.60)	<0.001
Serum total bilirubin level						
<0.8 mg/dL	1,365 (74.9)	7,561	32	423	1.00	
≥0.8 mg/dL	457 (25.1)	2,522	16	634	1.39 (0.76–2.54)	0.285
Serum total protein level						
>7.1 g/dL	1,362 (74.8)	7,480	33	441	1.00	
≤7.1 g/dL	460 (25.3)	2,603	15	576	1.18 (0.64–2.18)	0.601
Serum albumin level						
>4.1 g/dL	1,330 (73.0)	7,302	21	288	1.00	
≤4.1 g/dL	492 (27.0)	2,781	27	971	3.11 (1.75–5.53)	<0.001
Serum alpha-1 globulin level						
>0.2 g/dL	1,219 (66.9)	6,701	23	343	1.00	
≤0.2 g/dL	603 (33.1)	3,382	25	739	2.20 (1.24–3.89)	0.007
Serum alpha-2 globulin level						
>0.6 g/dL	495 (27.2)	2,814	11	391	1.00	
≤0.6 g/dL	1,327 (72.8)	7,269	37	509	1.30 (0.66–2.55)	0.450
Serum beta globulin level						
<1 g/dL	1,256 (68.9)	6,904	29	420	1.00	
≥1 g/dL	566 (31.1)	3,179	19	598	1.47 (0.82–2.63)	0.192
Serum gamma globulin level						
<1.4 g/dL	943 (51.8)	5,192	13	250	1.00	
≥1.4 g/dL	879 (48.2)	4,891	35	716	3.07 (1.62–5.81)	<0.001
Serum apolipoprotein A1 level						
<149 mg/dL	1,358 (74.5)	7,537	33	438	1.00	
≥149 mg/dL	464 (25.5)	2,546	15	589	1.64 (0.88–3.05)	0.116
Serum apolipoprotein B level						
>72 mg/dL	1,343 (73.7)	7,433	35	471	1.00	
≤72 mg/dL	479 (26.3)	2,650	13	491	1.17 (0.62–2.21)	0.636

+: Using the quartile of each seromarker as the cut-off point except AAR.

* : Age, gender, and study townships were included in Cox proportional hazards models.

Abbreviation: HCC, hepatocellular carcinoma; HBeAg, hepatitis B e antigen; Anti-HCV, antibodies against hepatitis C virus; HBV, hepatitis B virus; AST, aspartate aminotransferase; ALT, alanine aminotransferase; AAR, AST/ALT ratio; AFP, alpha-fetoprotein; GGT, gamma-glutamyltransferase.

### Risk Models Combining Different HCC Predictors

Three risk models combining different sets of HCC predictors with their assigned scores are shown in Table 2. Risk Model I was the “classical risk model” and included age, gender, serum ALT levels, and HBV seromarkers in the model. This set of predictors has been previously reported to have good validity in predicting the 3-, 5- and 10-year risk of HCC in patients with chronic hepatitis B. [14] Risk Model II included age, gender, and the liver-related seromarkers ALT, AAR, AFP, GGT, albumin, and alpha-1 globulin as predictors, but did not include HBV seromarkers. Risk Model III included HBV seromarkers in addition to the predictors included in Risk Model II. All predictors included in each model were statistically significant except gender. Considering that gender differences are usually observed in HBV-related HCC, it was still included in the three risk models with an assigned score of 1. The Pearson correlation coefficients among seromarkers included in the scoring models ranged from −0.34 to 0.29. Therefore, the effect of colinearity among these seromarkers was considered minimal.

**Table 2:** Multivariate-adjusted Hazard Ratios (HR), Regression Coefficients, and Assigned Scores for Hepatocellular Carcinoma Predictors in Three Risk Models.

Predictors		Hazard Ratio/Regression Coefficient/Assigned Score											
		Risk Model I				Risk Model II				Risk Model III			
		HR (95%CI)	β coefficient	Score	P-value	HR (95%CI)	β coefficient	Score	P-value	HR (95%CI)	β coefficient	Score	P-value
Age (5-year increments from 40 years old)		1.58 (1.35–1.86)	0.4601	1	<0.001	1.37 (1.16–1.61)	0.3110	1	<0.001	1.40 (1.18–1.66)	0.3355	1	<0.001
Gender	Female	1.00	Reference	0		1.00	Reference	0		1.00	Reference	0	
	Male	1.55 (0.65–3.71)	0.4372	1	0.317	1.59 (0.65–3.86)	0.4633	1	0.306	1.59 (0.64–3.96)	0.4634	1	0.320
ALT (U/L)	<28	1.00	Reference	0		1.00	Reference	0		1.00	Reference	0	
	≥28	3.06 (1.61–5.83)	1.1189	2	<0.001	4.65 (2.46–8.76)	1.5358	5	<0.001	2.68 (1.36–5.28)	0.9848	3	0.005
AAR	<1					1.00	Reference	0		1.00	Reference	0	
	≥1					9.04 (3.13–26.06)	2.2012	7	<0.001	7.19 (2.46–20.99)	1.9723	6	<0.001
AFP (ng/mL)	<5					1.00	Reference	0		1.00	Reference	0	
	≥5					4.71 (2.50–8.86)	1.5486	5	<0.001	3.65 (1.86–7.14)	1.2938	4	<0.001
GGT (U/L)	<41					1.00	Reference	0		1.00	Reference	0	
	≥41					3.50 (1.77–6.92)	1.2525	4	<0.001	3.44 (1.73–6.86)	1.2354	4	<0.001
Albumin (g/dL)	>4.1					1.00	Reference	0		1.00	Reference	0	
	≤4.1					3.13 (1.74–5.63)	1.1400	4	<0.001	2.54 (1.39–4.64)	0.9308	3	0.003
Alpha-1 globulin (g/dL)	>0.2					1.00	Reference	0		1.00	Reference	0	
	≤0.2					2.07 (1.15–3.72)	0.7266	2	0.015	1.94 (1.08–3.50)	0.6638	2	0.027
HBeAg negative and HBV DNA ≤10,000*		1.00	Reference	0						1.00	Reference	0	
HBeAg negative and HBV DNA >10,000*		4.11 (1.93–8.75)	1.4144	3						3.19 (1.46–6.98)	1.1608	3	0.004
HBeAg positive		14.50 (6.50–32.35)	2.6743	6						4.72 (1.99–11.19)	1.5524	5	<0.001

Abbreviation: HBeAg, hepatitis B e antigen; HBV, hepatitis B virus; ALT, alanine aminotransferase; AAR, aspartate aminotransferase/alanine aminotransferase ratio; AFP, alpha-fetoprotein; GGT, gamma-glutamyltransferase.

* : Serum HBV DNA level, copies per mL.

### Validity and AUROC of Three Risk Models

**Figure 2:**
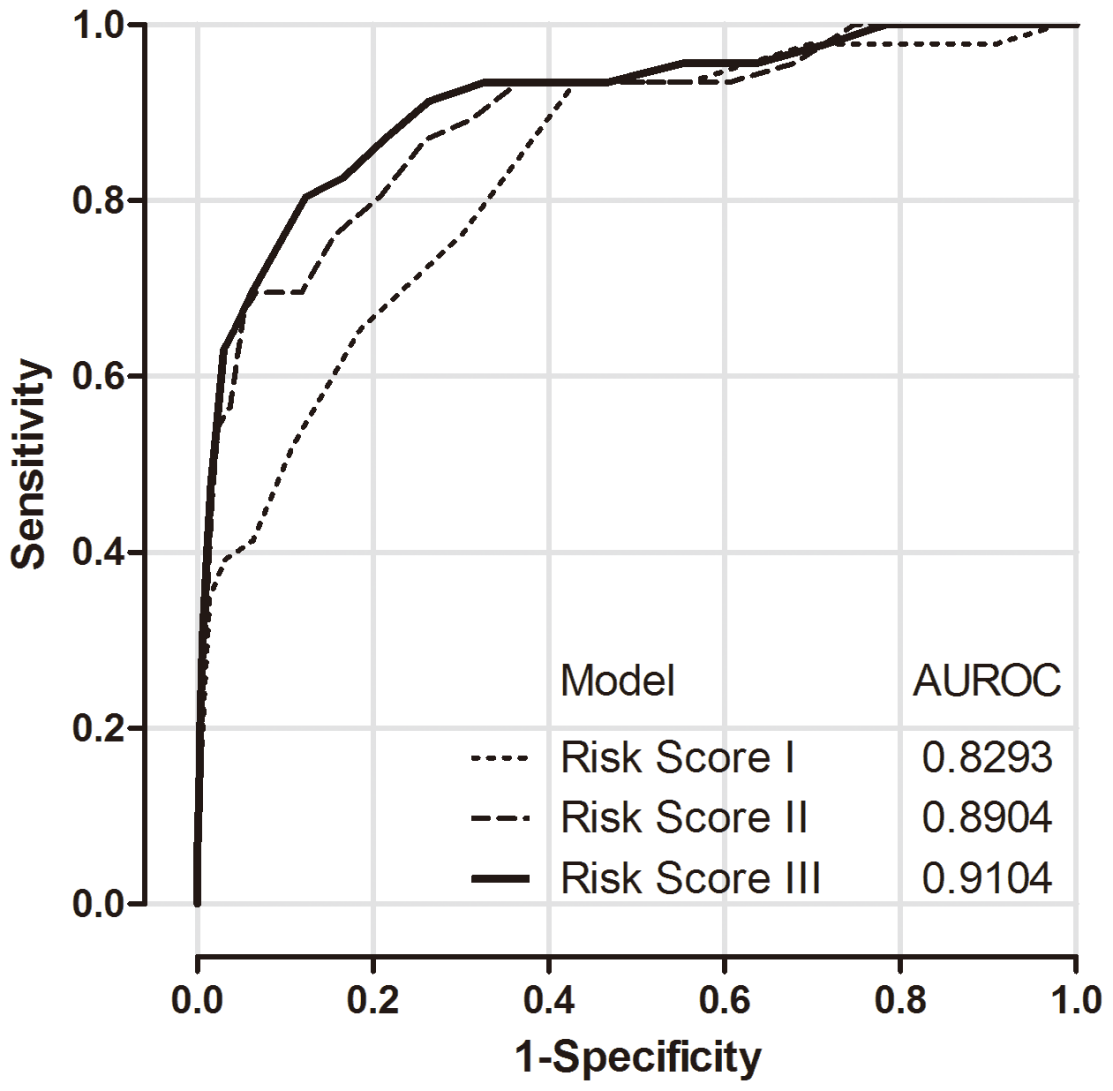
Receiver operating characteristic curves (ROCs) and areas under receiver operating characteristic curves (AUROCs). ROCs and AUROCs for the prediction of the 6-year incidence of hepatocellular carcinoma using sum scores of three risk models: The classical model (Risk Model I, dotted line), the model combining liver-related seromarkers without HBV seromarkers of HBeAg serostatus and serum HBV DNA level (Risk Model II, broken line), and the model combining liver-related seromarkers with HBV seromarkers (Risk Model III, solid line).

Figure 2 shows the ROC curves for predicting 6-year HCC risk using the total scores of the three risk models. Sum scores were derived by adding the scores assigned to all risk predictors included in each specific model. For example, when applying Risk Model III to a 62-years-old (score = 4) man (score = 1) with serum ALT levels of 90 U/L (score = 3), an AAR of 1.1 (score = 6), AFP of 3 ng/mL (score = 0), GGT of 29 U/L (score = 0), albumin of 4.7 g/dL (score = 0), and alpha-1 globulin of 0.3 g/dL (score = 0), as well as HBeAg-seronegative status with a serum HBV DNA level of 50,000 copies/mL (score = 3), the total score is 17.

The AUROC of Risk Model I was 0.83 for predicting 6-year HCC risk, which was consistent previous studies. [14] The best Youden index of Risk Model I was 0.50 for the 6-year prediction, while the positive likelihood ratio (LR+) and negative likelihood ratio (LR−) were 2.17 and 0.11, respectively. The AUROC, best Youden index, LR+, and LR− were 0.89, 0.63, 10.38, and 0.33, respectively for Risk Model II, as well as 0.91, 0.68, 6.52 and 0.22, respectively for Risk Model III. When defining the cut-off point by using the highest Youden index, the best cut-off total scores were set as 6 for Model I, 22 for Model II, and 19 for Model III. The corresponding sensitivity and specificity according to these cut-offs were 0.93 and 0.57 for Model I; 0.70 and 0.93 for Model II; and 0.80 and 0.88 for Model III, respectively. The predictability of the three risk models was considered very good to excellent with significant improvements when comparing the AUROC of Risk Model I with the AUROCs of Risk Model II (P = 0.01) and Risk Model III (p<0.001).

As most of the predictors included in Risk Model II and Risk Model III were also seromarkers for cirrhosis, whether or not the risk models could differentiate HCC from cirrhosis was further assessed. The student's t-test was used for comparing the means of total scores between cirrhotic patients with or without HCC, and between HCC participants with or without cirrhosis. For Risk Model II, the mean (95% CI) sum score was 15.1 (14.1–16.2) for 135 cirrhotic patients without HCC, and 24.4 (22.0–26.8) for 27 cirrhotic patients with HCC. For Risk Model III, the mean (95% CI) sum score was 14.9 (13.9–15.9) for 135 cirrhotic patients without HCC and 24.0 (21.9–26.2) for 27 cirrhotic patients with HCC. The differences in means were statistically significant (P<0.001) between cirrhotic patients with or without HCC for both Risk Model II and III. However, the mean sum score was not significantly different between HCC patients with and without cirrhosis for either Risk Model II (P = 0.403) or Risk Model III (P = 0.300). This suggests that both Risk Models had good HCC risk predictability, regardless of cirrhosis status.

Since liver-related seromarkers may change over time, another set of seromarkers in blood samples collected on dates different from baseline was compared to that of blood samples collected and analyzed to derive the risk scores. Among the 1,822 participants, 1,543 had another blood sample collected at a later time point (mean time interval: 314 days, median time interval: 356 days). Comparing sum scores of ALT, AAR, AFP, GGT, albumin, and alpha-1 globulin from Risk Model II at the two time points, there was no significant difference in total score based on a paired t-test (P = 0.77). Similar results were found for HCC cases (n = 35, mean time interval: 229 days, median time interval: 184 days, P = 0.87) and non-HCC cases (n = 1,508, mean time interval: 316 days, median time interval: 356 days, P = 0.79), respectively.

**Figure 3:**
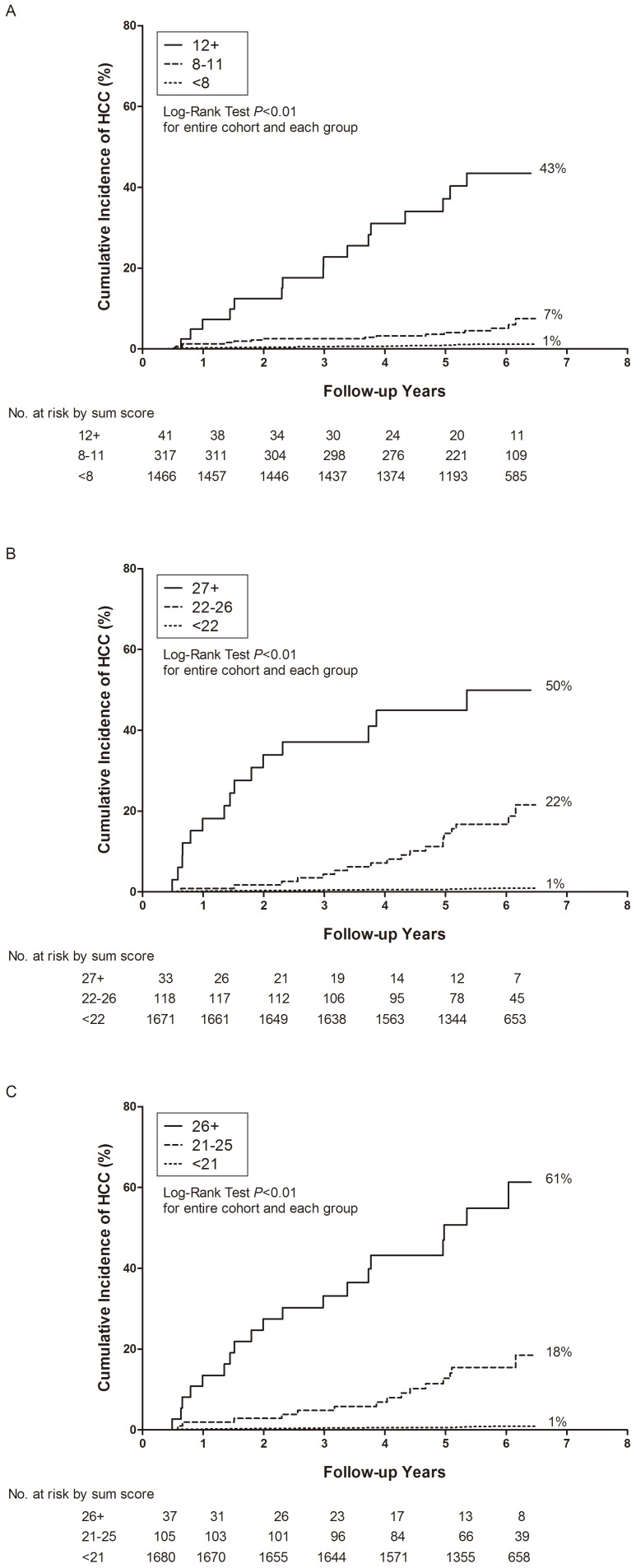
Cumulative incidence of hepatocellular carcinoma (HCC) by sum scores of each model at study entry. Classical model (Risk Model I, A), the model combining liver-related seromarkers without HBV seromarkers of HBeAg serostatus and serum HBV DNA level (Risk Model II, B), and the model combining liver-related seromarkers with HBV seromarkers (Risk Model III, C).

Figure 3 shows the cumulative incidence of HCC for participants classified into three groups using their sum scores from each risk model. Cut-off points of sum scores were set as 8 and 12 for Risk Model I, 22 and 27 for Risk Model II, and 21 and 26 for Risk Model III, according to the best stratification of HCC cases into three approximately equal groups. There were striking differences in cumulative HCC incidence during follow-up between the three groups with high, medium, and low sum scores, suggesting that the three Risk Models had an excellent discriminating capability to triage chronic HBV carriers with regards to their 6-year risk of HCC.

### Estimated HCC Incidence by Risk Model II and III

Table 3 shows the estimated 6-year cumulative HCC risk according to sum scores of Risk Model II and III. For Risk Model II, the estimated 6-year cumulative risk of HCC ranged from 0.01% for a total score of 0 to 96.71% for a total score of 34. For Risk Model III, the estimated 6-year cumulative risk of HCC ranged from 0.01% for a total score of 0 to 99.52% for a total score of 33. Both Risk Models had a wide range of estimated HCC risk, again suggesting their excellent discriminating capability.

**Table 3:** Estimated 6-year Incidence of Hepatocellular Carcinoma Using Risk Models Combining Liver-related Seromarkers without (Model II) and with (Model III) HBV Seromarkers of HBeAg Serostatus and Serum HBV DNA Level.

Risk Model II		Risk Model III	
Sum Score	Estimate Risk (%)	Sum Score	Estimate Risk (%)
0	0.01	0	0.01
1	0.01	1	0.01
2	0.02	2	0.02
3	0.02	3	0.02
4	0.03	4	0.03
5	0.04	5	0.04
6	0.06	6	0.06
7	0.08	7	0.09
8	0.11	8	0.12
9	0.14	9	0.17
10	0.20	10	0.24
11	0.27	11	0.33
12	0.36	12	0.46
13	0.50	13	0.65
14	0.68	14	0.90
15	0.92	15	1.26
16	1.26	16	1.76
17	1.71	17	2.46
18	2.33	18	3.42
19	3.16	19	4.75
20	4.29	20	6.58
21	5.81	21	9.08
22	7.85	22	12.46
23	10.55	23	16.98
24	14.12	24	22.92
25	18.76	25	30.52
26	24.69	26	39.91
27	32.09	27	50.96
28	41.03	28	63.08
29	51.37	29	75.19
30	62.61	30	85.76
31	73.89	31	93.46
32	84.00	32	97.79
33	91.80	33	99.52
34	96.71		

## Discussion

HBV-associated HCC remains a serious public health problem worldwide, especially in developing countries with lower income and inadequate medical resources. [26] In this study, we aimed to develop valid and easy-to-use HCC prediction models for chronic HBV carriers using liver-related seromarkers. In previous studies, various HBV seromarkers including serum HBV viral load, genotype, and mutants were used as predictors in HCC risk prediction models. [11]–[14] However, these seromarkers are not routinely tested in regular health examinations at most clinics. Therefore, we aimed to develop prediction models combining several liver-related seromarkers for HCC, even though we had already proposed a good prediction model (Risk Model I). In this study, we found that both models combining liver-related seromarkers with (Risk Model III) or without (Risk Model II) HBV seromarkers of serum HBV DNA level and HBeAg serostatus had good validity and discriminating capability to differentiate chronic HBV carriers with variable risks of developing HCC.

All of the liver-related seromarkers we analyzed are associated with liver diseases such as fibrosis, cirrhosis, jaundice, and fatty liver. They may reflect the status of the liver, such as liver cells injury, inflammation, necroinflammation, or proliferation, at the time of examination. AAR has been suggested as a fibrosis marker. By analyzing these 13 liver-related seromarkers along with the AAR, we first tried to find out candidate predictors that had significant associations with HCC. Then, we further selected predictors according to the results of multivariate analysis and biological rationale. Finally, liver-related seromarkers included in the models were ALT, AAR, AFP, GGT, albumin, and alpha-1 globulin. All of them were significantly associated with HCC in multivariate analyses. Among the six predictors, ALT, AAR, AFP, GGT, and albumin are widely used. Although Alpha-1 globulin is not commonly used, it had an independent and significant association with HCC in this study, and is also available in clinical practice. In addition, both ALT and albumin have been found to be HCC predictors in chronic hepatitis B patients. [12]–[14] AFP is a well-known HCC seromarker, and AAR is a cirrhotic marker that may reflect progressive liver functional impairment if it equals 1 or higher. [22] Serum GGT mostly comes from liver; it has been found to be useful for the diagnosis of HCC in patients with low AFP levels or at a relatively early stage. [27] The deficiency of alpha-1-antitrypsin, the main (∼90%) protein of the alpha-1 globulins, has been found to be associated with an increased risk for liver damage, cirrhosis and HCC. [28].

In the long natural history of chronic hepatitis B, risk predictors of HCC may change over time. Some HCC predictors are more appropriate for long-term prediction, while some are better short-term predictors. Usually, liver fibrosis and cirrhosis occur sequentially before the development of HCC in chronic hepatitis B patients. During the hepatopathogenic progression, however, various seromarkers are changing dynamically. These seromarkers may thus be categorized as long-term and short-term risk predictors. When the onset of HCC is closer, useful predictors will shift from the long-term predictors, such as HBeAg serostatus and serum HBV DNA level, to the short-term predictors, which may be surrogate variables of severe fibrosis or even cirrhosis. Both ALT and GGT are inflammatory markers; AAR and albumin are associated with liver fibrosis; AFP is associated with liver cell proliferation; and decreased alpha-1 globulin proteins may either be a sign of alpha-1 antitrypsin deficiency related to the pathogenesis of HCC, or reflect the reduced production of required proteins due to impaired liver function. All are biomarkers that reflect the disease status of the liver, and may be referred to as short-term predictors. However, ALT is also a long-term predictor since it is related to the hosts immunoreactivity to HBV.

Thus, the importance of HBeAg serostatus and serum HBV DNA levels in the prediction of HCC risk may decrease when chronic hepatitis B progresses from the immune tolerance phase, through the immune clearance phase, to the residual phase. HBeAg serostatus and HBV DNA levels had less additional predictability for the short-term risk of HCC if seromarkers of liver necroinflammation and fibrosis/cirrhosis were also taken into consideration. As HBeAg serostatus and serum HBV DNA level are the most important long-term predictors of HCC, antiviral therapy to lower viral load has a very significant impact on the reduction of HCC risk, especially for those patients without severe fibrosis or cirrhosis. The efficacy of therapeutic intervention for hepatitis B has been assessed and proven. Although sustained HBV DNA suppression is rarely associated with HBV clearance, it results in reduced rates of liver complications, including HCC and the progression of cirrhosis. [17] Though the importance of HBeAg serostatus and serum HBV DNA level shows is minor for the 6-year prediction of HCC, they still provide useful information for the continued care of chronic hepatitis B patients. The findings of this study suggest that in addition to antiviral therapy to lower HBV viral loads, other interventions to reverse fibrosis and cirrhosis are also important for the reduction of HCC risk.

Besides HBV seromarkers, age, gender, and liver cirrhosis are usually considered as important predictors in HCC risk models. [11], [12] Liver cirrhosis is a precursor to HCC. Each year, 2–8% of patients with HBV-related cirrhosis, either compensated cirrhosis or decompensated, may further develop HCC. [29] Cirrhosis status was not included as a predictor in our models because a confirmatory diagnosis of cirrhosis using liver biopsy was not feasible in a community-based cohort. Furthermore, most of the liver-related seromarkers used in our models are associated with liver stiffness and may be considered as surrogate biomarkers for cirrhosis. [30].

Since participants in this community-based prospective study were healthier than patients in hospital-based studies, most participants would be classified as “normal” for these liver-related seromarkers if current clinical cut-off points were applied. Therefore, in this study, the cut-off points of these seromarkers were set by their first or third quartiles instead of the normal limits routinely used in clinical practice, such as 40 U/L for ALT and 20 ng/mL for AFP. Indeed, the revised cut-off points of these seromarkers were found to be significantly associated with an increased risk of subsequent HCC. We used binary variables to reduce the influence of occasional fluctuations because the scoring was based on the dichotomous classification of “positive” or “negative” instead of exact measurements of liver-related seromarkers. In Risk Models I and III, serum HBV DNA level and HBeAg serostatus were combined to classify participants into three groups: 1) HBeAg-seronegatives with serum HBV DNA level ≤10,000 copies/mL, 2) HBeAg-seronegatives with serum HBV DNA level >10,000 copies/mL, and 3) HBeAg-seropositives, as almost all HBeAg-seropositives had very high serum levels of HBV DNA. The cut-off point of 10,000 copies/mL was used for serum HBV DNA levels according to our previous observation that serum HBV DNA levels seldom rebound again once they have spontaneously decreased ≤10,000 copies/mL. [1] The combination of several seromarkers was considered adequate for deriving a stable risk estimation.

A specific aim of this study was to develop an evidence-based risk score to provide clinicians with the expected HCC risk for a patient infected with chronic hepatitis B. Developing such a score may help patient consultation and clinical management. The validity of the prediction models was evaluated using the AUROC as shown in Figure 2, and by assessing their discriminatory capability as shown in Figure 3. Furthermore, we provided an estimate of 6-year HCC risk according to total scores, which makes it easy for clinicians to obtain a patient’s 6-year risk of HCC and then give the patient clinical advice accordingly. Based on these findings, we suggest that chronic HBV carriers be tested for their liver-related seromarkers at their first clinical visit, and that our Risk Model II be used to calculate their probability of developing HCC in 6 years. Both HBeAg serostatus and serum HBV DNA level may be further tested to derive Risk Model III for the refinement of HCC risk prediction. Those who have high total scores should be further assessed for (1) whether or not they can be treated with antivirals or immune moderators to interrupt the progression of chronic hepatitis B to severe fibrosis, cirrhosis or even HCC; (2) how frequently they should be monitored by abdominal ultrasonography to detect HCC at a very early stage; and (3) what healthier lifestyles they should maintain.

There are some limitations to be considered if these risk models are to be applied to chronic HBV carriers in other populations. Firstly, almost all study participants were infected with HBV during early childhood and experienced the immune tolerance phase in this analysis. Whether or not the findings in this study may be applicable to patients infected in adulthood needs further elucidation. Secondly, participants enrolled in this study were middle aged adults. Therefore, the risk models may not apply to younger chronic HBV carriers. Thirdly, only seromarkers at study entry were included in this analysis, and whether seromarkers during follow-up are also important in risk prediction also deserves further elucidation. Lastly, all participants were infected with HBV genotype B and/or C, and whether or not the natural history of chronic HBV infection is the same for genotypes A, D or others also remains to be clarified.

In conclusion, we found that liver-related seromarkers may be combined to predict HCC risk of chronic hepatitis B patients, and two risk models incorporating liver-related seromarkers with and without HBV seromarkers were developed for the prediction of the 6-year risk of HCC. The models were found to have high validity and discriminating capability for the identification of high-risk groups for HCC. These risk models were accurate, inexpensive and easy-to-use. However, further external validation of the risk models is needed if they will be applied to chronic hepatitis B patients in different populations.
